# Burden of healthcare-associated infections in tertiary care hospitals in six countries of the Gulf Cooperation Council: A point prevalence survey

**DOI:** 10.1017/S0950268826101964

**Published:** 2026-10-14

**Authors:** Majid M. Alshamrani, Aiman El-Saed, Fayssal Farahat, Mohammed Al Zunitan, Mohammed Abalkhail, Sarah Alfayez, Najiba Mohamed Abdulrazzaq, Afaf Merza Mohamed, Abdulrhman Ibrahim Mohana, Nawal Al Kindi, Jameela Ali Al-Ajmi, Kelly Schrapp, Rana H. Almaghrabi, Hussam Alobaida, Amal Al Tai, Fatimah Saad Alshahrani, Zahir O. Babiker, Najla Ibrahim Alrabei, Maher Alharbi, Khaled Al Sager, Ahmad Subhi, Wafa Al Nasser, Abdulmajid Al Arfaj, Syed Nazeer, Maida Balila, Alaa Alqurashi, Nouf Alzuhayri, Salman Alhajri, Rasha Alfawaz, Sami Almudarra, Pasi Penttinen

**Affiliations:** 1 Infection Prevention and Control Department, King Abdulaziz Medical City, Ministry of National Guard Health Affairs, Riyadh, Saudi Arabia; 2 King Saud bin Abdulaziz University for Health Sciences, Ministry of National Guard Health Affairs, Riyadh, Saudi Arabia; 3 King Abdullah International Medical Research Center,Ministry of National Guard Health Affairs, Riyadh, Saudi Arabia; 4 Al Kuwait Hospital Dubai, Dubai, United Arab Emirates; 5 Salmanyia Medical Complex, Manama, Bahrain; 6 Public Health Authority, Riyadh, Saudi Arabia; 7 Khoula Hospital, Muscat, Oman; 8 Hamad Medical corporation, Doha, Qatar; 9 Jaber Al Ahmed hospital, Kuwait, Kuwait; 10 Prince Sultan Military Medical City, Riyadh, Saudi Arabia; 11 King Fahad Medical City, Riyadh, Saudi Arabia; 12 Royal hospital, Muscat, Oman; 13 King Saud University Medical City, Riyadh, Saudi Arabia; 14 Sheik Shakhbout Medical City, Abu Dhabi, United Arab Emirates; 15 King Abdulaziz Medical City, King Saud bin Abdulaziz University for Health Sciences, King Abdullah International Medical Research Center, Ministry of National Guard Health Affairs, Jeddah, Saudi Arabia; 16 King Abdullah Specialized Children’s Hospital, Ministry of National Guard Health Affairs Riyadh, Saudi Arabia; 17 Al Qassimi Hospital, Sharjah, United Arab Emirates; 18 Imam Abdulrahman Bin Faisal Hospital, Dammam, Saudi Arabia; 19 King Abdulaziz Hospital, Al Ahsa, Saudi Arabia; 20 Prince Mohammed Bin Abdulaziz Hospital, Al Madinah, Saudi Arabia; 21 Mediclinic City Hospital, Dubai, United Arab Emirates; 22 Gulf Center for Disease Prevention and Control, Gulf Health Council, Saudi Arabia

**Keywords:** Gulf Cooperation Council, healthcare-associated infection, hospitals, pathogens, point prevalence survey

## Abstract

A cross-sectional study using point prevalence survey (PPS) methodology was conducted on May 20, 2024, at 18 tertiary care hospitals located in the 6 Gulf Cooperation Council (GCC) countries to assess the prevalence of healthcare-associated infections (HAIs) and associated pathogens. The measured outcome was the presence of active HAIs in different hospital units. During the PPS day, 651 HAI events were detected among 580 patients after reviewing the information of 6826 patients. The prevalence of patients with HAIs was 8.5% (95% CI 7.8%–9.2%). The prevalence was higher in older patients (11.8%, 95% CI 10.2%–13.4%), in intensive care units (24.6%, 95% CI 21.5%–27.8%), and in specialty care areas (12.7%, 95% CI 9.8%–15.7%). The most frequent type of HAIs was pneumonia (27.8%), followed by bloodstream infection (25.0%), urinary tract infection (10.9%), and skin infection (9.7%). Device-associated HAIs represented 15.4% of all HAIs, with ventilator-associated pneumonia, central line-associated bloodstream infection, and catheter-associated urinary tract infection being the main types. Approximately 61.9% of HAIs were microbiologically confirmed. Out of them, 65.3% were caused by Gram-negative bacteria and 31.8% were caused by multidrug-resistant pathogens. The findings confirm that the high HAI burden in GCC hospitals is higher in high-resource countries but lower in lower/middle-resource countries.

## Introduction

Healthcare-associated infections (HAIs) remain a major challenge for patients, healthcare service providers, and facilities [1]. They expose patients to a twofold to threefold increase in mortality and more than threefold increase in the length of stay [2]. Additionally, the diagnosis of HAI is associated with more than a twofold increase in healthcare expenses [3]. The increase in healthcare cost is even augmented in HAI caused by pathogens resistant to common antimicrobials [4, 5]. Traditional HAI surveillance primarily focuses on device-associated infections and surgical site infections (SSIs) [6]. Therefore, a point prevalence survey (PPS) is considered the practical tool to provide a comprehensive estimate of the burden of HAIs, including both traditionally and non-traditionally surveyed types of HAI [7]. Additionally, conducting repeated PPSs is essential for monitoring HAI trends, identifying areas of improvement, and assessing the effectiveness of currently implemented preventive measures [8].

The estimated burden of HAI, as assessed by PPS, varies significantly across different geographic regions. HAI has been estimated between 3% and 7% in American and European hospitals, respectively [9, 10]. On the other hand, the prevalence of HAI was much higher in low-resource countries, ranging between 9% and 32% [11–13]. The Gulf Cooperation Council (GCC) region is conventionally regarded as a high-resource region [14]. Nevertheless, the infection rates of individual device-associated HAIs were intermediate between high-resource and low-resource countries [15, 16]. Only a few studies used PPS to estimate the overall burden of HAIs in GCC member states [17–20], while most of the studies covered one country [18–20]. There are insufficient studies addressing all GCC member states or analysing risk factors associated with HAIs. Improved understanding of the true burden of HAIs in GCC member states will enable the Gulf Centre for Disease Control and Prevention (CDC) and policymakers to set targets for preventive interventions and optimize resource utilization. Furthermore, such data would be important in updating surveillance plans across the hospitals in the region. The objective of this study was to assess the prevalence of HAIs and associated pathogens in tertiary care hospitals in six GCC countries.

## Methods

### Setting

This study was carried out at 18 tertiary care hospitals located in six GCC countries. These included ten hospitals in Saudi Arabia, three in the United Arab Emirates, two in Oman, and one hospital each in Bahrain, Kuwait, and Qatar. Included hospitals have bed capacities ranging from 200 beds to 1 200 beds, with a total bed capacity of more than 12 000 beds. The Gulf CDC served as the coordinating centre for the study, overseeing the study protocol, data entry, and data validation processes. The Gulf-CDC-IPC Expert Hub, based at the Infection Prevention and Control Programme at King Abdulaziz Medical City (KAMC), Ministry of National Guard Health Affairs in Riyadh, was responsible for training infection control professionals who carried out the actual data collection. Furthermore, the Expert Hub was responsible for overall data management, analysis, and interpretation.

### Design

This study was designed as a cross-sectional PPS conducted on 20 May 2024. The protocol followed established frameworks developed by the European Centre for Disease Prevention and Control (ECDC) [7] and the American National Healthcare Safety Network (NHSN) [21].

### Study population

This study included patients of all ages who had been admitted on the day of the PPS to any eligible unit in the participating hospitals. Non-eligible units were units that do not accept overnight admission, such as outpatient clinics including outpatient dialysis, emergency department, and same-day surgery units. Patient inclusion criteria were further divided into numerator and denominator criteria. The numerator criteria included patients with an active HAI and/or receiving antimicrobial therapy on the day of PPS. The denominator criteria included patients admitted to a ward/unit before or at 8 a.m. and not discharged from the ward/unit during the PPS day. They also included those who were temporarily off the unit for diagnostic investigations or procedures.

### Sample size

Previous studies done in GCC countries estimated the HAI point prevalence between 7% and 11% [17, 18]. Assuming a prevalence of 9% and a confidence limit of 1%, at least 3137 hospital beds would be required to detect the assumed prevalence at a 95% significance level. The required sample size was distributed between countries according to expected capacity and population size; 50% for Saudi Arabia and 10% for each of the other five countries. As the hospitals were the first recruitment unit, minor variation in such allocation was allowed. The sample size was calculated using the OpenEpi application [22].

### Sampling technique

Hospitals were chosen conveniently from each country. Hospitals were tertiary care hospitals, had more than 100 beds, had electronic patient chart systems, and had a well-established infection control department. Sampling technique of beds (patients) was not applicable; all eligible patients from the selected tertiary care hospitals were included.

### Data collection

Data were collected using the REDCap program [23]. Specialized data collection forms were created: a unit form and a patient form. The unit form was filled out for each unit included. The form specified the country, hospital name, and unit name and listed the age and gender of all admitted patients. In case of neonatal Intensive care units (ICUs), birth weight was additionally recorded. The patient form was filled out only in the presence of active HAI and/or receiving antimicrobial therapy. It included data such as country, hospital name, unit name, and HAI-related information – age, gender, comorbidity, date of infection, type of infection, possible source of infection, presence of device, culture results, type of specimen, and associated pathogens.

### Training

Prior to the study, data collectors at the participating hospitals underwent training on the study protocol, including the study design, eligibility criteria, algorithms for case identification, standards for infection triggers, definitions of HAIs, procedures for data validation, and using the data collection application (REDCap). The training consisted of several interactive sessions in addition to access to consultation with an epidemiologist before, during, and after the PPS day. Although the study was conducted in one day, resolving issues related to the diagnosis of infection and completing data entry took up to four weeks after the PPS day.

### Case finding algorithm

On the PPS day, the start date and time of the survey were recorded, data on unit speciality and number of beds were gathered, a patient list for each unit was obtained, patients admitted after 8:00 a.m. were excluded, and denominator data (unit form) from all patients in the units before 8:00 a.m. were collected. For each patient, the presence of infection was initially checked using the triggers suggesting the presence of infection. They included fever (>38 °C), receiving antimicrobials (antibiotics or antifungals), leucopenia or leucocytosis (<4 k/μL or ≥ 12 k/μL), hypotension (SBP < 90 mmHg), tachypnoea (>20/min), altered mental status (Glasgow Coma Scale <15), invasive device (central line, urinary catheters, mechanical ventilator), and recent surgery (within the last 30/90 days). The final determination of infection and the completion of all other required information were finalized in the following few days after the PPS day.

### Outcome definition

The primary outcome of the study was the presence of an active infection, which occurred more than two days from the admission date. Active HAIs were defined as infections that met NHSN surveillance definition criteria, with other signs or symptoms of infection present on the PPS day or ongoing antimicrobial treatment for an HAI on the PPS day.

### Validation

Data were validated at multiple levels to ensure accuracy and consistency. Adjudication of preliminary diagnosis was performed by reviewing information among data collectors and experts at participating hospitals. Challenging scenarios were verified with an epidemiologist before, during, and after the PPS day. Data entry restrictions were set by the REDCap program, and final validation was completed before data analysis by resolving discrepancies and missing information.

### Statistical analysis

Collected data were entered in a pre-prepared REDCap project. Data were exported to the Statistical Package for the Social Sciences software (SPSS Version 29.0. Armonk, NY: IBM Corp) before data management, including verification, completeness, cleaning, and transformation. The categorical variables were presented as frequency and percentage. For the continuous variables, they were initially tested for normality distribution and presented using mean and standard deviation (SD) if normally distributed or median and interquartile range (IQR) if not. The prevalence of HAIs was calculated by dividing the identified HAI events by the total number of patients reviewed. The prevalence rates were then stratified by age, gender, hospital, and unit. Differences in the prevalence of HAIs in different groups were examined using a chi-squared test or Fisher’s exact test as appropriate. All P-values were two-tailed. A *P*-value <0.05 was considered significant. SPSS was used for all statistical analyses.

## Results

During the PPS day, 651 HAI events were detected among 580 patients after reviewing the information of 6 826 patients. The largest proportion of reviewed patients was from Saudi Arabia (*n* = 3 595, 52.7%), followed by Oman (*n* = 843, 12.3%), the UAE (*n* = 740, 10.8%), Qatar (*n* = 696, 10.2%), Bahrain (*n* = 583, 8.5%), and Kuwait (*n* = 369, 5.4%). The median (IQR) duration between admission and the development of infection was 11 (2–37) days. The characteristics of patients with infection are shown in Table 1. The mean age (±SD) was 46.4 ± 28.1 years, with 12.9% of the patients being one year or less and 33.1% of the patients being above 65 years. Approximately 57.8% of the patients were males and 42.2% were females. The most frequent location of admission was wards (52.4%), followed by ICUs (30.3%), speciality care areas such as oncology, haematology, and organ transplantation units (10.7%), and neonatal ICU (5.2%). In neonatal ICU, the median (IQR) birth weight was 1 850 (965–2 615) g. The most frequent birth weight groups were ≤1 000 g (36.7%), >2 500 g (30.0%), and 1 501–2 500 g (23.3%). The most frequent comorbidities were diabetes (31.9%), hypertension (31.6%), heart disease (27.4%), respiratory disease (17.8%), malignancy (14.5%), and neurologic disease (13.4%).

**Table 1.** Characteristics of patients with healthcare-associated infections during the point prevalence survey in GCC countries, May 2024 (*N* = 580) Table 1 long description.

	Number	Percentage
Age (years)		
Mean ± SD	46.4 ± 28.1	
≤1	75	12.9
2–17	51	8.8
18–65	262	45.2
>65	192	33.1
Gender		
Male	335	57.8
Female	245	42.2
Location		
ICU	176	30.3
NICU	30	5.2
Speciality care area	62	10.7
Ward	304	52.4
ER > 1 day	8	1.4
Birth weight groups of neonates (g)		
Median (IQR)	1850 (965–2615)	
≤1 000	11	36.7
1 001–1 500	3	10.0
1 501–2 500	7	23.3
>2 500	9	30.0
Total	30	100.0
Comorbidity		
Diabetes	185	31.9
Hypertension	183	31.6
Heart disease	159	27.4
Respiratory	103	17.8
Malignancy	84	14.5
Neurologic	78	13.4
Dialysis	50	8.6
Renal no dialysis	49	8.4
Immunosuppression	40	6.9
Gastrointestinal	34	5.9
Trauma	26	4.5
Hepatobiliary	24	4.1
Burn	11	1.9
Others	63	10.9
Day to infection, median (IQR)	11 (2–37)	

Data were presented as number and percentage, unless mentioned otherwise. SD, standard deviation; IQR, interquartile range. Birth weight groups were collected only in neonatal intensive care units.

As shown in Table 2, the prevalence of patients with at least one HAI event was 8.5% (95% CI 7.8%–9.2%). On the other hand, the prevalence of HAI events (considering all events in case a patient has multiple HAIs) was 9.5 events (8.8–10.2) per 100 patients. The prevalence of patients with at least one HAI event was significantly higher in older (>65 years) patients (11.8%, 95% CI 10.2%–13.4%), in patients admitted to ICU (24.6%, 95% CI 21.5%–27.8%), and in patients admitted to speciality care areas (12.7%, 95% CI 9.8%–15.7%). The prevalence was non-significantly higher in males than females (9.3% versus 7.6%). In neonatal ICUs, the prevalence was highest in those weighted ≤1 000 g (9.9%) and lowest in those weighted 1 001–1 500 g (5.7%). Country-wise, the prevalence was significantly higher in Oman, Saudi Arabia, and the United Arab Emirates (10.9%, 9.5%, and 9.2%, respectively) than in Bahrain and Qatar (3.8% and 4.2%, respectively).

**Table 2.** Infection rates (95% CI) during the point prevalence survey in GCC countries, 20 May 2024 Table 2 long description.

	Number of patients reviewed	Number of patients with at least one HAI event	Prevalence of patients with at least one HAI event (Percentage)	Number of HAI events considering multiple events in the same patient	Prevalence of HAI events considering multiple events in the same patient (HAIs per 100 patients)
Overall	6 826	580	8.5% (7.8%–9.2%)	651	9.5 (8.8–10.2)
Age (years)					
≤1	761	75	9.9% (7.7%–12.0%)	82	10.8 (8.6–13.0)
2–17	808	51	6.3% (4.6%–8.0%)	55	6.8 (5.1–8.5)
18–65	3 628	262	7.2% (6.4%–8.1%)	297	8.2 (7.3–9.1)
>65	1 629	192	11.8% (10.2%–13.4%)	217	13.3 (11.7–15.0)
Gender					
Male	3 607	335	9.3% (8.3%–10.2%)	369	10.2 (9.2–11.2)
Female	3 219	245	7.6% (6.7%–8.5%)	282	8.8 (7.8–9.7)
Location					
ICU	715	176	24.6% (21.5%–27.8%)	215	30.1 (26.7–33.4)
NICU	369	30	8.1% (5.3%–10.9%)	31	8.4 (5.6–11.2)
Speciality care area	488	62	12.7% (9.8%–15.7%)	68	13.9 (10.9–17.0)
Ward	5 101	304	6.0% (5.3%–6.6%)	328	6.4 (5.8–7.1)
ER > 1 day	153	8	5.2% (1.7%–8.8%)	9	5.9 (2.2–9.6)
Birth weight groups (NICU)					
≤1000	111	11	9.9% (4.4%–15.5%)	11	9.9 (4.4–15.5)
1001–1500	53	3	5.7% (0.0%–11.9%)	3	5.7 (0.0–11.9)
1501–2500	95	7	7.4% (2.1%–12.6%)	8	8.4 (2.8–14.0)
>2500	101	9	8.9% (3.4%–14.5%)	9	8.9 (3.4–14.5)
Total	360	30	8.3% (5.5%–11.2%)	31	8.6 (5.7–11.5)
Country					
Bahrain	583	22	3.8% (2.2%–5.3%)	23	3.9 (2.4–5.5)
Kuwait	369	26	7.0% (4.4%–9.7%)	27	7.3 (4.7–10.0)
Oman	843	92	10.9% (8.8%–13.0%)	99	11.7 (9.6–13.9)
Qatar	696	29	4.2% (2.7%–5.7%)	31	4.5 (2.9–6.0)
Saudi Arabia	3 595	343	9.5% (8.6%–10.5%)	389	10.8 (9.8–11.8)
United Arab Emirates	740	68	9.2% (7.1%–11.3%)	82	11.1 (8.8–13.3)

**Figure 1.**
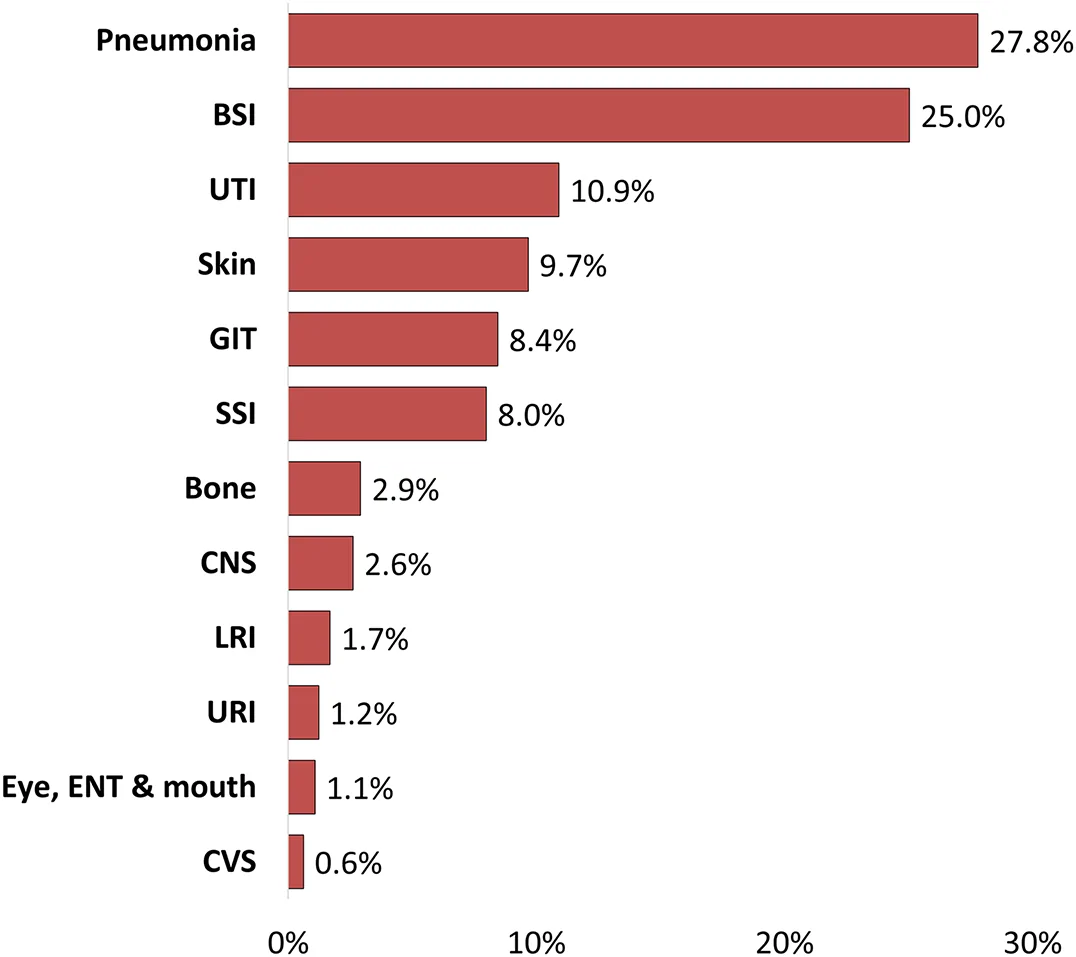
Frequency of types of infection diagnosed during the point prevalence survey in GCC countries, 20 May 2024 (*N* = 666). *Abbreviations:* BSI, bloodstream infection; UTI, urinary tract infection; Skin, skin and soft tissue infection; GIT, gastrointestinal infection; SSI, surgical site infection; CNS, central nervous system infection; LRI, lower respiratory infection; URL, upper respiratory infection; ENT, ear, nose, and throat infection; CVS, cardiovascular infection. Figure 1 long description.

As shown in Figure 1, the most frequent type of HAI was pneumonia (27.8%), followed by bloodstream infection (BSI, 25.0%), urinary tract infection (UTI, 10.9%), skin and soft tissue infection (9.7%), and gastrointestinal infection (GIT, 8.4%). Table 3 shows the breakdown of these infections by hospital locations and patient groups. Pneumonia and BSI were the two most common infections (>50%) in all patient groups. They were more common in ICUs than wards (68.7% versus 43.2%, *P* < 0.001) but not different by patient groups (*P* = 0.0825). UTI, skin and soft tissue infection, GIT, and SSI together were more common in adult and paediatric patients compared with neonatal patients (40.1% and 29.3% versus 9.7%, respectively, *P* < 0.001). They were also more common in wards than ICUs (46.9% versus 20.7%, *P* < 0.001).

**Table 3.** Frequency of types of infection by location and patient groups examined during the point prevalence survey in GCC countries, 20 May 2024 (*N* = 651) Table 3 long description.

	Hospital locations		Patient groups		
	ICUs	Wards	Adults	Paediatrics	Neonates
	*N* = 246, %	*N* = 405, %	*N* = 521, %	*N* = 99, %	*N* = 31, %
Pneumonia	100 (40.7)	81 (20.0)	142 (27.3)	28 (28.3)	11 (35.5)
BSI	69 (28.0)	94 (23.2)	128 (24.6)	29 (29.3)	6 (19.4)
UTI	17 (6.9)	54 (13.3)	60 (11.5)	10 (10.1)	1 (3.2)
Skin	17 (6.9)	46 (11.4)	56 (10.7)	6 (6.1)	1 (3.2)
GIT	11 (4.5)	44 (10.9)	45 (8.6)	9 (9.1)	1 (3.2)
SSI	6 (2.4)	46 (11.4)	48 (9.2)	4 (4.0)	0 (0.0)
Bone	1 (0.4)	18 (4.4)	17 (3.3)	2 (2.0)	0 (0.0)
CNS	8 (3.3)	9 (2.2)	12 (2.3)	2 (2.0)	3 (9.7)
LRI	6 (2.4)	5 (1.2)	7 (1.3)	1 (1.0)	3 (9.7)
URI	4 (1.6)	4 (1.0)	2 (0.4)	6 (6.1)	0 (0.0)
Eye, ENT, and mouth	5 (2.0)	2 (0.5)	1 (0.2)	1 (1.0)	5 (16.1)
CVS	2 (0.8)	2 (0.5)	3 (0.6)	1 (1.0)	0 (0.0)

Abbreviations: BSI, bloodstream infection; UTI, urinary tract infection; Skin, skin and soft tissue infection; GIT, gastrointestinal infection; SSI, surgical site infection; CNS, central nervous system infection; LRI, lower respiratory infection; URL, upper respiratory infection; ENT, ear, nose, and throat infection; CVS, cardiovascular infection.

**Figure 2.**
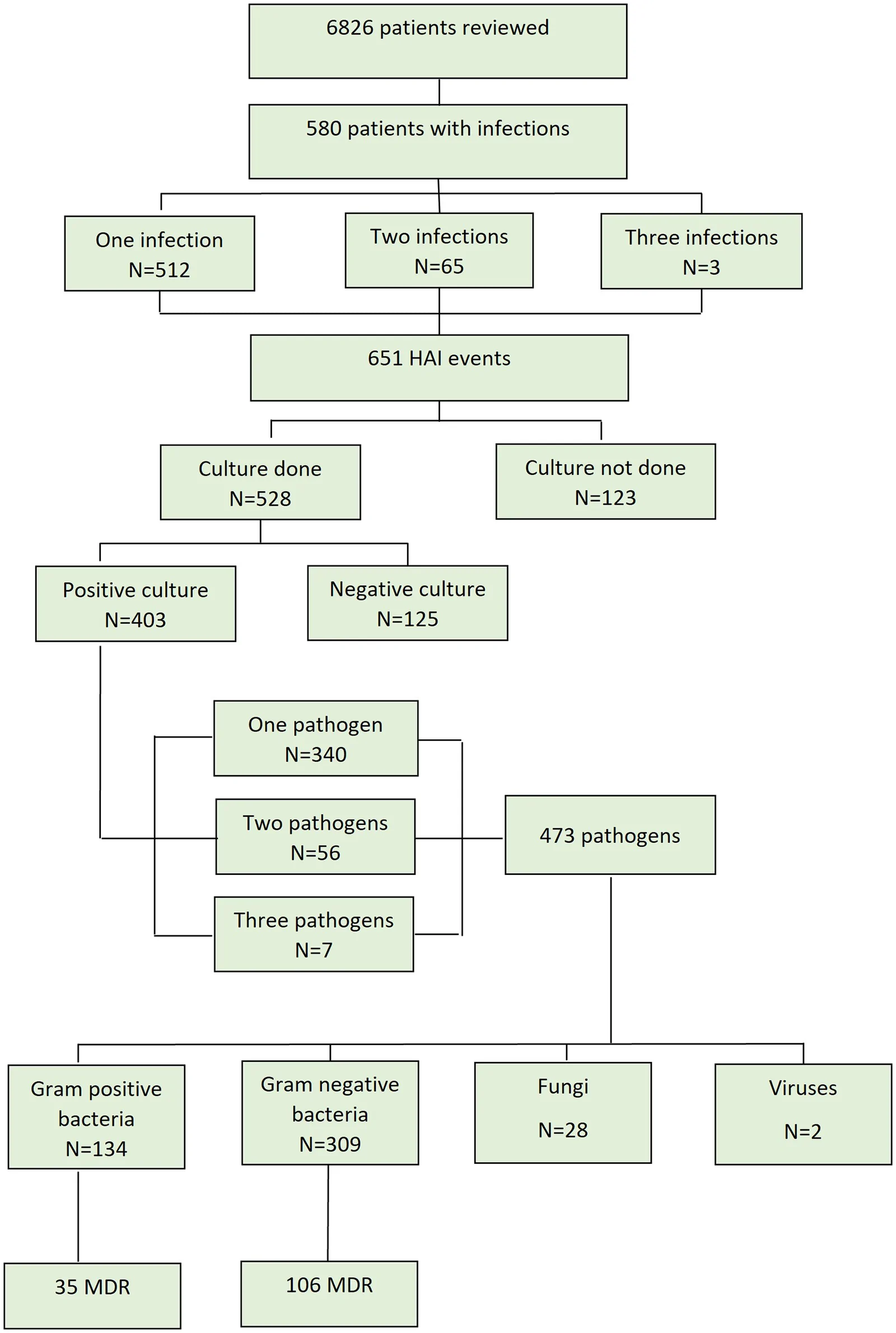
Flow chart of the HAI events and associated cultures, pathogens, and MDR pathogens. Figure 2 long description.

Table 4 highlights the characteristics of HAI events diagnosed during the PPS day. Additionally, the flow chart (Figure 2) details the numbers for events, cultures, pathogens, and multidrug-resistant (MDR) pathogens. The majority of HAI diagnoses were identified as a single infection per patient (78.6%). The source of the majority of HAI events was believed to be the current hospital (88.6%). Approximately 15.4% of the HAI events were device-associated; mainly ventilator-associated pneumonia (VAP) (45.0%) and central line-associated bloodstream infection (CLABSI) (37.0%), and to a lesser extent catheter-associated urinary tract infection (CAUTI) (18.0%). Cultures were performed for the majority of HAI events (81.1%). The most common type of specimen was blood (36.5%), followed by respiratory specimens (19.6%), urine (15.1%), and wound (13.8%). Approximately 76.3% of the cultures were positive. Most of the cultures had one pathogen (82.9%), while 14.7% and 2.4% of the cultures had two or three pathogens, respectively. Multiple pathogens (mainly two) per culture were frequently seen in wound (25.4%) and respiratory (19.8%) specimens but were lacking in rectal specimens. Approximately 61.9% of HAIs were microbiologically confirmed. Out of them, 65.3% were caused by Gram-negative bacteria and 28.3% were caused by Gram-positive bacteria. Approximately 31.8% of HAIs were caused by MDR pathogens, with higher rates of MDR contribution to skin and soft tissue infections (54.2%), bone infections (43.8%), and UTIs (36.4%). As shown in Figure 2, the most frequent pathogens reported were *Klebsiella* spp. (19.2%), *Pseudomonas* spp. (17.1%), *Staphylococcus aureus* (11.0%), and *Escherichia coli* (10.6%). These four pathogens represented more than half (57.9%) of all pathogens reported.

**Table 4.** Characteristics of infections diagnosed (*N* = 651) during the point-prevalence survey in GCC countries, 20 May 2024 Table 4 long description.

Number of infections diagnosed (*N* = 651)		
One infection per patient	512	78.6%
Two infections per patient	65 × 2	20.0%
Three infections per patient	3 × 3	1.4%
Possible source of infection (*N* = 651)		
Current hospital	577	88.6%
Previous hospital	74	11.4%
Presence of device (*N* = 651)		
No	410	63.0%
Yes	241	37.0%
Device-associated HAI (*N* = 651)		
No	551	84.6%
Yes	100	15.4%
Types of device-associated HAI (*N* = 100)		
VAP	45	45.0%
CLABSI	37	37.0%
CAUTI	18	18.0%
Type of specimen (*N* = 608)		
Blood	222	36.5%
Respiratory	119	19.6%
Urine	92	15.1%
Wound	84	13.8%
Stool	14	2.3%
Rectal	2	0.3%
Others	75	12.3%
Culture done (*N* = 651)		
No	123	18.9%
Yes	528	81.1%
Positive culture (*N* = 528)		
No	125	23.7%
Yes	403	76.3%
Microbiologically confirmed HAIs (*N* = 651)		
No	248	38.1%
Yes	403	61.9%
Number of retrieved pathogens (*N* = 473)		
One pathogen per culture	340	71.9%
Two pathogens per culture	56 × 2	23.7%
Three pathogens per culture	7 × 3	4.4%
Type of retrieved pathogens (*N* = 473)		
Gram-positive bacteria	134	28.3%
Gram-negative bacteria	309	65.3%
Fungi	28	5.9%
Virus	2	0.4%
MDR bacterial pathogens (*N* = 443)		
No	302	68.2%
Yes	141	31.8%

**Figure 3.**
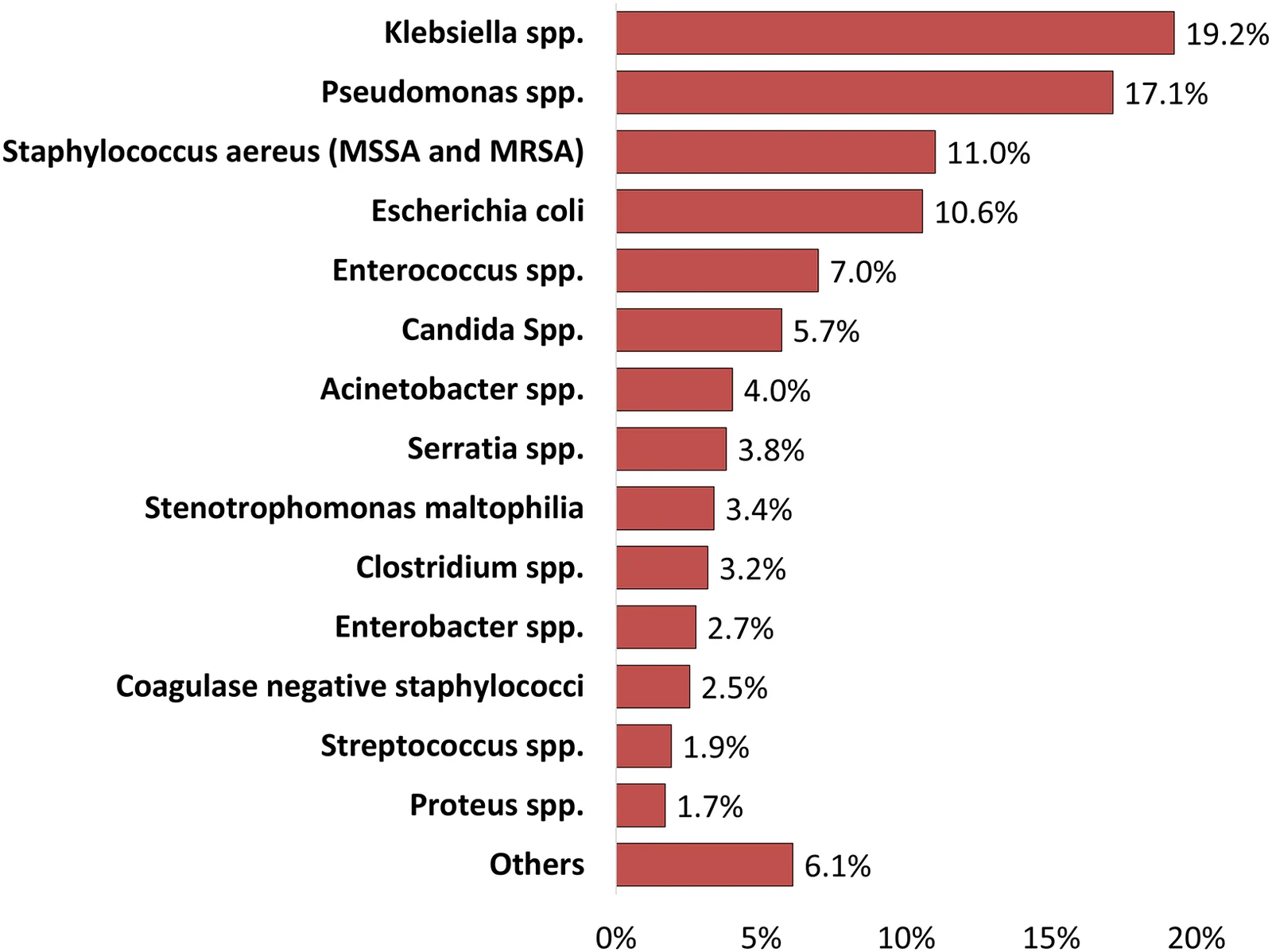
Frequency of pathogens associated with infections (*N* = 500) diagnosed during the point prevalence survey at GCC countries, 20 May 2024. *Abbreviations:* MSSA, methicillin-susceptible *Staphylococcus aureus*; MRSA, methicillin-resistant *Staphylococcus aureus.* Figure 3 long description.

## Discussion

The current study examined the burden of HAIs in 18 tertiary care hospitals located in six GCC countries. The prevalence of patients with at least one HAI event was 8.5% while the prevalence of HAI events (considering multiple events in the same patient) was 9.5%. The current figures were within the range of the findings of the few multicentre studies done previously in the GCC region, ranging between 6.5% and 11.8%. For example, a PPS study done in 11 secondary and tertiary care hospitals in five GCC countries, in addition to Egypt and Lebanon, estimated that the prevalence of patients with at least one HAI event was 11.8% [17]. Another study done in six National Guard hospitals across Saudi Arabia estimated a prevalence of 6.5% for patients with at least one HAI event and 6.8% for the prevalence of HAI events overall [18]. Obviously, the above two studies were either dependent on or heavily driven by data from Saudi Arabia, which represented 40% of the included patients.

Interestingly, the prevalence of HAI in the GCC region is considered higher than that of high-resource countries [9, 10, 24] but lower than global figures and those of low-resource countries [11–13]. This is likely reflecting the investment in healthcare systems and the maturation of infection control programmes [25, 26]. For example, large PPS studies in high-resource countries estimated the prevalence of HAI at 7% in >1 300 hospitals in 28 European countries [9], 3.2% in ~200 hospitals in the USA [10], and 7.7% in four hospitals in Japan [24]. Additionally, the prevalence of HAI in reviews and meta-analysis studies based on data from low-resource countries was estimated between 12.8% and 27.0% in African countries [11, 12] and between 9.0% and 12.7% in Southeastern Asian countries [11, 13]. It should be noted that the global prevalence of HAI in a large meta-analysis was estimated at 14.1% (12.7% to 15.6%) [11].

As expected, pneumonia was the most frequent type of HAI, followed by BSI, UTI, skin infection, and GIT infections. Interestingly, these five types of HAIs represented more than 80% of all HAI diagnoses in the current study. Pneumonia has been invariably shown as the primary HAI infection in previous studies done in high-resource countries [9, 10] and GCC countries [17, 18], while SSI has been shown as the primary HAI infection in low-resource countries [12]. In all settings, the above five types of HAIs were among the most frequent types of HAI [9, 10, 12, 17, 18]. Additionally, pneumonia and BSI were more common in ICUs, while UTI, skin and soft tissue infection, GIT, and SSI were more common in wards [8]. This is probably reflecting the distribution of patients’ age and gender, disease severity, length of stay, and the use of devices [8].

Device-associated HAIs represented 15% of the HAI events detected in the current study, with VAP, CLABSI, and CAUTI as the main types. Previous studies showed that device-associated HAIs represent between 19% and 28% of all HAIs, with CAUTI, CLABSI, and VAP identified as the main types [9, 10, 18, 27]. However, studies were variable regarding the relative frequency of the three types of infections. For example, CAUTI [9, 18], VAP [13, 27], or both [10] were all reported as the most frequent device-associated HAIs. Since ventilators and central lines are largely used in ICUs and urinary catheters are more utilized in wards [27], the relative frequency of different types of device-associated HAIs is likely influenced by the percentage of ICU patients included in the PPS. The higher VAP and CLABSI in the current study may be related to the high percentage of ICU patients in the current study (15.8%) compared with many previous studies (4% to 15%) [9, 10, 18, 27]. The current findings underscore the importance of regular PPS to monitor non-device infections, which represent the majority of HAIs but are not covered by routine surveillance [8].

Several characteristics and risk factors of HAI events in the current study were similar to previous evidence. For example, the risk of developing HAI events in the current study was significantly higher with admission to ICU or speciality care area and in patients with old age. This finding is consistent with earlier studies that linked HAI occurrence with admission to ICU or haematology wards, older age > 65 years, presence of devices, especially mechanical ventilators, and antimicrobial consumption [28–30]. The average hospital stay before the development of infection in the current study was 11 days, which was very close to what has been reported by the European (12 days) and American (13 days) hospitals [9, 10].

More than 60% of HAI events in the current study were microbiologically confirmed. The frequency of microbiologically confirmed HAIs was similar to those reported in Europe (61%) and the US (70%) [9, 10]. The frequency of laboratory-confirmed HAIs was very variable between different types of infection [9, 10]. For example, less than 50% of pneumonia cases are laboratory-confirmed, while 100% of BSI are laboratory-confirmed in American hospitals [10]. Two-thirds of the pathogens in the current study were Gram-negative bacteria, with the most frequent pathogens being *Klebsiella* spp., *Pseudomonas* spp., and *E. coli.* This was very close to studies done in the GCC region, where 60% or more of HAI events are caused by Gram-negative bacteria, especially the above three pathogens [31–33]. Internationally, studies show significant variability in the prevalence of pathogens, with some reporting higher levels of *E. coli* or Acinetobacter than those observed in this research [9–11, 13]. Differences in the predominant pathogens are probably related to the relative frequency of different types of infection. The MDR pathogens in the current study were responsible for more than 30% of microbiologically confirmed HAI events. This was very close to studies done in the GCC region, where between 20% and 34% of HAI events were caused by MDR pathogens [31–33]. European hospitals reported 40% resistance in pathogens causing HAI events, with generally higher Gram-positive resistance in Western studies [9, 10].

The current study is considered the largest HAI PPS study from the GCC region. The study was preceded by training on the study protocol, and a scientific hub was assigned to address queries or resolve potential issues before and during the study. Data were collected using a standardized electronic system and were validated and analysed centrally. Nevertheless, the participating hospitals were considered large elite tertiary care hospitals, which may limit the generalization of the findings to smaller or non-tertiary care hospitals in the region.

## Conclusions

The prevalence of patients with at least one HAI event was 8.5% in a sample of tertiary care hospitals in GCC countries. Pneumonia was the most frequent type of HAI, followed by BSI, UTI, skin infection, and GIT infections. Device-associated HAIs represented 15% of the HAI events, with VAP, CLABSI, and CAUTI being the main types. Two-thirds of HAI events were microbiologically confirmed, with more than 30% of them caused by MDR pathogens. The findings indicate that the burden of HAIs in GCC hospitals is higher than in high-resource countries but lower than in lower/middle-resource countries. These results can help in setting surveillance priorities, with more focus on wards and common HAIs not covered by regular surveillance. Additionally, these data can establish a baseline for future PPS studies aiming to monitor the effectiveness of the infection control practices in the region.

## Data Availability

The datasets used and analysed during the current study are available from the corresponding author on reasonable request and after approval of the IRB.

## Table 1 Long description

The mean age (±SD) was 46.4 ± 28.1 years, with 12.9% of the patients were one year or less, and 33.1% of the patients were above 65 years. Approximately 57.8% of the patients were males and 42.2% were females. The most frequent location of admission was wards (52.4%), followed by ICUs (30.3%), speciality care areas such as oncology, haematology, and organ transplantation units (10.7%), and neonatal ICU (5.2%). In neonatal ICU, the median (IQR) birth weight was 1850 (965–2,615) g. The most frequent birth weight groups were ≤ 1,000 g (36.7%), >2,500 g (30.0%), and 1,501–2,500 g (23.3%). The most frequent comorbidities were diabetes (31.9%), hypertension (31.6%), heart disease (27.4%), respiratory disease (17.8%), malignancy (14.5%), and neurologic disease (13.4%).

Navigate back to Table 1.

## Table 2 Long description

The prevalence of patients with at least one HAI event was 8.5% (95% CI 7.8%–9.2%). On the other hand, the prevalence of HAI events (considering all events in case a patient has multiple HAIs) was 9.5 events (8.8–10.2) per 100 patients. The prevalence of patients with at least one HAI event was significantly higher in older (>65 years) patients (11.8%, 95% CI 10.2%–13.4%), in patients admitted to ICU (24.6%, 95% CI 21.5%–27.8%), and in patients admitted to speciality care areas (12.7%, 95% CI 9.8%–15.7%). The prevalence was non-significantly higher in males than females (9.3% versus 7.6%). In neonatal ICUs, the prevalence was highest in those weighted ≤1,000 g (9.9%) and lowest in those weighted 1,001–1,500 g (5.7%). Country wise, the prevalence was significantly higher in Oman, Saudi Arabia, and United Arab Emeritus (10.9%, 9.5%, and 9.2%, respectively) than Bahrain and Qatar (3.8% and 4.2%, respectively).

Navigate back to Table 2.

## Figure 1 Long description

As shown in Figure 1, the most frequent type of HAI was pneumonia (27.8%), followed by bloodstream infection (BSI, 25.0%), urinary tract infection (UTI, 10.9%), skin and soft tissue infection (9.7%), and gastrointestinal infection (GIT, 8.4%).

Navigate back to Figure 1.

## Table 3 Long description

Pneumonia and BSI were the most common two infections (>50%) in all patient groups. They were more common in ICUs than wards (68.7% versus 43.2%, p < 0.001) but not different by patient groups (p = 0.0825). UTI, skin and soft tissue infection, GIT, and SSI together were more common in adult and pediatric patients compared with neonatal patients (40.1% and 29.3% versus 9.7%, respectively, p < 0.001). They were also more common in wards than ICUs (46.9% versus 20.7%, p < 0.001).

Navigate back to Table 3.

## Table 4 Long description

The majority of HAI diagnoses were identified as a single infection per patient (78.6%). The source of the majority of HAI events was believed to be the current hospital (88.6%). Approximately 15.4% of the HAI events were device-associated; mainly VAP (45.0%) and CLABSI (37.0%), and to a lesser extent CAUTI (18.0%). Cultures were performed for the majority of HAI events (81.1%). The most common type of specimen was blood (36.5%), followed by respiratory specimens (19.6%), urine (15.1%) and wound (13.8%). Approximately, 76.3% of the cultures were positive. Most of the cultures had one pathogen (82.9%), while 14.7% and 2.4% of the cultures had two or three pathogens (respectively). Multiple pathogens (mainly two) per culture were frequently seen in wound (25.4%) and respiratory (19.8%) specimens but were lacking in rectal specimens. Approximately 61.9% of HAIs were microbiologically confirmed. Out of them, 65.3% were caused by Gram-negative bacteria and 28.3% were caused by Gram-positive bacteria. Approximately 31.8% of HAIs were caused by MDR pathogens, with higher rates of MDR contribution to skin and soft tissue infections (54.2%), bone infections (43.8%), and UTIs (36.4%).

Navigate back to Table 4.

## Figure 2 Long description

The flow chart details the number for events, cultures, pathogens, and MDR pathogens. During the PPS day, 651 HAI events were detected among 580 patients after reviewing the information of 6,826 patients. Out of the 651 events, 403 were microbiologically confirmed. Out of them, 309 were caused by Gram-negative bacteria and 141 were caused by multidrug-resistant pathogens.

Navigate back to Figure 2.

## Figure 3 Long description

The most frequent pathogens reported were Klebsiella spp. (19.2%), Pseudomonas spp. (17.1%), Staphylococcus aereus (11.0%), and Escherichia coli (10.6%). These four pathogens represented more than half (57.9%) of all pathogens reported.

Navigate back to Figure 3.
